# Exploring the Structural Diversity and Biotechnological Potential of the Rhodophyte Phycolectome

**DOI:** 10.3390/md23010008

**Published:** 2024-12-26

**Authors:** Éllen F. Rodrigues, Flavia Alves Verza, Felipe Garcia Nishimura, Renê Oliveira Beleboni, Cedric Hermans, Kaat Janssens, Maarten Lieven De Mol, Paco Hulpiau, Mozart Marins

**Affiliations:** 1 Postgraduate Program in Environmental Technology, University of Ribeirão Preto/UNAERP, Ribeirão Preto 14096-900, SP, Brazil; efrodrigues@unaerp.br; 2 Biotechnology Unit, University of Ribeirão Preto/UNAERP, Ribeirão Preto 14096-900, SP, Brazilfelipegnishi@hotmail.com (F.G.N.); rbeleboni@unaerp.br (R.O.B.); 3 Bioinformatics Knowledge Center (BiKC), Cluster Life Sciences, Campus Brugge Station, Howest University of Applied Sciences, Spoorwegstraat 4, 8200 Brugge, Belgium; cedric.hermans@howest.be (C.H.); kaat.janssens@howest.be (K.J.); 4 Centre for Industrial Biotechnology and Biocatalysis (InBio.be), Department of Biotechnology, Faculty of Bioscience Engineering, Ghent University, Coupure Links 653, 9000 Ghent, Belgium; maarten.demol@ugent.be; 5 Algastech Aquiculture, Research and Development, Ubatuba 11695-722, SP, Brazil

**Keywords:** Rhodophyta, red seaweed, phycolectome, lectins

## Abstract

Lectins are non-covalent glycan-binding proteins found in all living organisms, binding specifically to carbohydrates through glycan-binding domains. Lectins have various biological functions, including cell signaling, molecular recognition, and innate immune responses, which play multiple roles in the physiological and developmental processes of organisms. Moreover, their diversity enables biotechnological exploration as biomarkers, biosensors, drug-delivery platforms, and lead molecules for anticancer, antidiabetic, and antimicrobial drugs. Lectins from Rhodophytes (red seaweed) have been extensively reported and characterized for their unique molecular structures, carbohydrate-binding specificities, and important biological activities. The increasing number of sequenced Rhodophyte genomes offers the opportunity to further study this rich source of lectins, potentially uncovering new ones with properties significantly different from their terrestrial plant counterparts, thus opening new biotechnological applications. We compiled literature data and conducted an in-depth analysis of the phycolectomes from all Rhodophyta genomes available in NCBI datasets. Using Hidden Markov Models capable of identifying lectin-type domains, we found at least six different types of lectin domains present in Rhodophytes, demonstrating their potential in identifying new lectins. This review integrates a computational analysis of the Rhodophyte phycolectome with existing information on red algae lectins and their biotechnological potential.

## 1. Introduction

Marine biomes occupy approximately 70% of the biosphere, harbor many different species, and are important sources of bioactive molecules. Within these marine biomes, macroalgae—classified into green algae (Chlorophyta), brown algae (Phaeophyta), and red algae (Rhodophyta), based on their pigment production [1]—are sessile, photosynthetic eukaryotic organisms. These organisms form diverse and abundant groups, ranging from unicellular to macroscopic structures. Their composition is influenced by many environmental factors, such as pH, temperature, and dissolved inorganic nitrogen, to which they are exposed [2]. Macroalgae provide important services to the marine ecosystem, such as food, shelter, and habitat for other species. They are notable for contributing significantly to the total primary productivity of temperate, arctic, and tropical systems around the world [3]. Not only do they significantly shape marine ecosystems, but they also possess a tremendous portfolio of natural products and proteins with bioactive properties, covered in various articles and literature reviews, i.e., antimicrobial, antiviral, antitumor, and agglutination activities [4,5,6,7,8]. Unsurprisingly, macroalgae have been harvested from wild environments and farmed for food or biotechnological purposes.

Among macroalgae, Rhodophyta holds great potential. Various species of Rhodophyta are commercially cultivated in tropical countries due to their simple cultivation process, rapid growth rate, and high productivity. This makes them a crucial source of income for numerous coastal communities [9]. They are often used as food sources or to produce carrageenan, a sulfated polysaccharide with a naturally active hydrocolloid that has important applications in the food industry [10,11]. Additionally, Rhodophyta also possesses many other valuable molecules with promising biotechnological applications, such as alkaloids, terpenoids, polyethers, polyketides, proteins and peptides [12]. Extensive research has focused on relatively small molecules, but macromolecules such as proteins, glycoproteins, and polysaccharides are also promising candidates for such functional agents as they play critical roles in many biological processes. Among these, lectins are particularly notable for their diverse functions [13]. Lectins are found in microorganisms, plants, and animals, and they bind reversibly and specifically to carbohydrates in biopolymers without altering their structure [14,15]. These proteins are present in many species of algae and perform essential functions, including cell adhesion, pathogen recognition, and growth regulation [16,17]. They can agglutinate erythrocytes due to their ability to specifically bind to carbohydrates, such as sugars or sugar residues found on the cell surface [18]. Initially treated as hemagglutinins (when the sugar specificity is unknown) or fusion proteins composed of carbohydrate-binding domains (rarely characterized as hemagglutinins), lectins were originally isolated from plant extracts [19]. However, later studies have shown that lectin families are widely distributed in nature and can also be found in humans, animals, microorganisms, and algae [20]. The first report of the presence of these proteins in marine algae was made in 1966 [21], and since then, many researchers have sought to unravel the biological activities of marine algae lectins.

Lectins are now regarded as proteins involved in supporting enzymatic reactions in the synthesis and degradation of glycans, specifically directed to parts of the glycan structures [22]. This binding specificity of lectins is crucial for their biological functions, where they participate as mediators in the protein–carbohydrate recognition stage in different biological events such as cellular communication, host defense, fertilization, cellular development, and parasitic infection [23,24].

In this study, we extensively reviewed published research on Rhodophyta lectins, focusing on their prevalence and diversity within this phylum and their potential applications in the biotechnological sector. Additionally, we scanned all publicly available Rhodophyta genomes with Hidden Markov Models (HMMs) using HMMER 3.4 to uncover the coding sequences and occurrence of known and novel lectins, thereby completing our overview of the Rhodophyta phycolectome.

## 2. Lectin Families and Their Protein Characteristics

**Figure 1:**
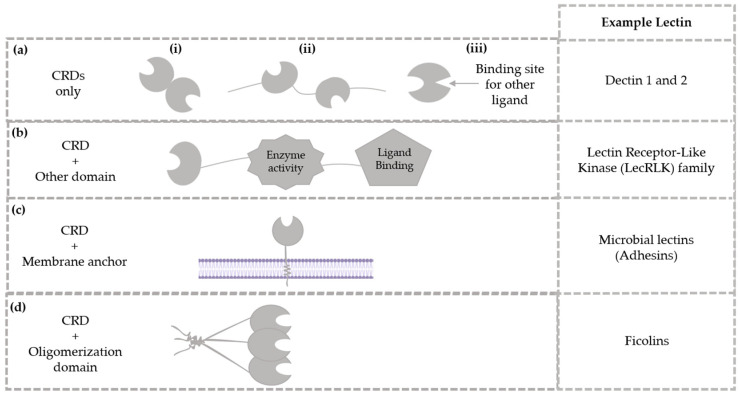
A schematic illustrating the arrangements of carbohydrate recognition domains (CRDs) in lectins. Lectins can contain (**a**) only CRDs, (**b**) CRDs in conjunction with other types of functional domains, (**c**) CRDs associated with membrane anchors, or (**d**) CRDs with oligomerization domains. It is also possible for a single lectin to contain all of these additional domains. Adapted from Taylor et al. [25], Saijo et al. [29], Sun et al. [30], Siebs et al. [31], Matsushita [32].

Naturally occurring glycan-binding proteins are present in all living organisms. They can be categorized into two main groups: lectins and sulfated glycosaminoglycan-binding proteins (GAG-binding proteins). Proteins capable of binding sulfated GAGs (such as heparan, chondroitin, dermatan, and keratan sulfates) utilize clusters of positively charged amino acids to interact with specific arrangements of carboxylic acid and sulfate groups along the GAG chains. Most of these proteins are evolutionarily unrelated [25]. Lectins, on the other hand, have been classified into evolutionarily related protein families characterized by their ability to bind specific carbohydrates such as mannose, galactose, lactose, N-acetylglucosamine, N-acetylgalactosamine, fucose, and rhamnose [26]. With the development of gene transcription analysis and the ability to interpret the structure of polypeptide chains in lectin molecules, an additional classification layer based on the similarity of amino acid sequences and protein folding is possible. The domains are responsible for the carbohydrate-binding specificity, called carbohydrate recognition modules or domains (CRDs); each has a unique amino acid sequence, a characteristic polypeptide fold structure, and a structural binding site [27]. Generally, CRDs are protein segments that fold independently and often act individually from other catalytic domains in the protein [28]. Besides substrate specificity, lectins can be divided into various classes based on the protein architecture. For example, in some cases, proteins consist solely of CRDs, as observed in dectin-1 and dectin-2 (Figure 1a) [29]. These may include lectins with linked subunits (Figure 1a(i)), those with tandem lectin domains within the polypeptide chain (Figure 1a(ii), and lectins possessing multiple distinct glycan-binding sites (Figure 1a(iii)). Conversely, other lectins incorporate additional domains that impart enzymatic activity, as seen in LecRLK family members (Figure 1b) [30]. Some lectins are equipped with membrane-anchoring domains, exemplified by microbial lectins, such as adhesins (Figure 1c) [31]. Others feature oligomerization-facilitating domains, as in ficolins (Figure 1d) [32]. An overview of these diverse lectin types is provided in Figure 1.

In recent years, various studies have been conducted to catalog the lectin composition from marine organisms, such as cyanobacteria, algae, invertebrates, and fish, and to clarify their bioactive and physiological properties [33]. In these organisms, lectins play important roles in several biological processes, such as cell–cell interactions, modulation of the immune response, and pathogen recognition. The properties of lectins from marine organisms depend on the species from which they are derived [34]. However, the current classification of lectins is complex, with different types originating from either their discovery source (i.e., ricin), substrate specificity (i.e., galectins), or specific protein folds (i.e., F-fold) [35,36,37]. Here, we will discuss lectin types associated with marine seaweed based on specific protein domains or folds.

Calcium-binding-type lectins or CTLs are a large group of extracellular proteins composed of at least one carbohydrate recognition or CTL-like domain with diverse functions. Initially characterized by a double-loop (‘loop-in-a-loop’) structure, the second loop has proven to be absent in compact CTLs. The loops are often evolutionarily conserved along with two disulfide bridges at the bases of the loops and hydrophobic and polar interactions to stabilize the structure [27]. Various classes and subclasses have been introduced for CTLs. While they are known as calcium-binding lectins, as they have a calcium-binding domain in their structure [38], not all CTLs are Ca^2+^-dependent [39].

F-type lectins or FTLs are characterized by a fucose recognition domain that exhibits a novel jellyroll fold (F-type fold), carbohydrate and calcium-binding sequence motifs, and a nominal specificity for L-fucose [40], a non-reducing terminal sugar found in pro- and eukaryotic glycans [41]. Many FTL isoforms are typically expressed in a single individual, arising from genetic mechanisms for ligand recognition diversification [42]. The architecture of FTLs can vary significantly, with single or multiple fucose recognition domains in tandem or interspersed with other distinct domains.

Galectin-type lectins or GTLs, formerly also known as S-type lectins because of their dependency on disulfide bridges for stabilization, have an affinity for carbohydrates containing lactose or N-acetylgalactosamine [43]. They are a class of Ca^2+^-independent lectins that bind to β-galactosides through a specific CRD-binding sequence. The galectin’s CRD is composed of about 140 residues containing a two antiparallel β-sheet sandwich with the shape of a closing hand. The carbohydrate-binding site is present in a groove in the S-sheet side of the sandwich, and the β-galactoside recognition core motif is located by sheets S4, S5, and S6 [44]. Despite their structural conservation, galectins exhibit remarkable functional diversity [45]. Three groups of galectins have been identified to date: those with only one CRD, a tandem repeat type containing two peptide-linked CRDs, and the chimera type, comprising an N-terminal Pro- and Gly-rich domain linked to the C-terminal CRD [46].

H-type lectins, or HTLs, are a relatively new group of lectins that share structural homology and primarily bind to N-acetylgalactosamine or GalNAc [47]. Initially identified in invertebrates such as snails, these lectins protect fertilized eggs from bacterial infections. Research on a coral lectin known as SLL-2 has shown that this protein plays a role in the symbiotic relationship between corals and microalgae. In slime molds, H-type lectins are crucial for social behavior. Additionally, members of the H-type lectin family have been successfully used in the diagnostics of various types of cancers.

Jacalin-type lectins (JTLs) were first identified in the albumin fraction of the seeds of jack fruit (*Artocarpus integrifolia*) [48], which named this group of lectins and from which the first jacalin lectins were cloned [49]. Recently, proteins with jacalin-related lectin domains have become a focal point in research on plant resistance responses. A meta-analysis of fusion proteins containing these domains across various kingdoms has revealed a wide array of partner domains, including kinases and toxins [50]. Unique to JTLs is the type I β-prism fold, called the jacalin-like lectin fold, which exists as three-fold symmetry with each domain containing a four-stranded Greek key motif [51]. The jackfruit jacalin is a homotetrameric lectin with a molecular mass of about 66 kDa. In the literature, only one JTL was uncovered from Rhodophyta, namely, the lectin griffithsin from *Griffithsia* sp. [52].

Legume-type lectins, or LTLs, are a broad range of lectins initially associated with plants. However, many LTLs have been identified in animals as well, such as calnexin and calreticulin. Legume-type lectins have also been found in yeast and red algae [53]. In general, they share large similarities up until the tertiary structure, yet their quaternary structure is dynamic and variable to allow flexibility regarding an array of carbohydrate ligands [37].

Malectins and malectin-like domain-containing lectins constitute a recently described group of conserved disaccharide-binding proteins of about 190 amino acid residues, which exhibit structural similarity to carbohydrate-binding modules (CBM) of bacterial glycosylhydrolases [54]. They were first reported in *Xenopus laevis* but later were found to be a highly conserved endoplasmic reticulum (ER) lectin in the animal kingdom, including humans. The malectin family is classified under the galactose-binding domain-like superfamily with proteins having one or more β-sandwich domains and a jellyroll topology [55].

Ricin-type lectins or RTLs, found in the seeds of the castor bean plant (*Ricinus communis*), are some of the best-known lectins as the first lectin to be discovered was ricin and RTLs are often highly toxic to many organisms, including humans [56]. The conserved structure of RTLs is comprised of an A- and a B-chain, synthesized as a single polypeptide, yet cleaved post-translationally. These chains are covalently linked by one or more disulfide bridges to form a monomer, although dimerization often occurs to create multimers [57]. RTLs contain an R-type carbohydrate-binding domain that is evolutionarily conserved between animal, plant, and bacterial lectins. This R-type domain shows a three-lobed shape that is a β-trifoil structure. In most RTLs, only one or two lobes show carbohydrate-binding affinity. In the case of ricin, two R-type carbohydrate binding domains are present on the B-chain in tandem [58].

## 3. The Rhodophyte Lectome

Marine algae are a rich source of lectins belonging to various classes. However, next to the lectins described in the literature, many more can be distilled from genomes in public databases. Therefore, in this Section, we investigated Rhodophyte genomes of NCBI Datasets for the presence of lectins. Hanschen et al. [59] have highlighted a concerning trend of deteriorating quality of genome assembly, gene annotation, and overall genome completeness in algal genomics. As of September 2024, among the 65 publicly available genomes of rhodophytes, indeed, many exhibited issues such as very low N50 values, a high number of scaffolds, lack of annotation, or even contamination. Some species, like *Galdieria sulphuraria*, had up to 10 different assemblies available. To provide a reliable overview of the Rhodophyte lectome, the highest quality and most well-annotated red algae genomes were selected from NCBI Datasets [60]. Fifteen Rhodophyta genomes were chosen for analysis using twenty-six HMMs (Supplementary Tables) from the Pfam protein family database to identify lectin domains [61]. HMM hits were compared against the annotated protein sequences in the NCBI Genome dataset for each species and were further analyzed using batch CD-search [62] and BLASTp against the RefSeq protein database. Only significant, complete matches with homologs in the RefSeq proteins were included in this review. Table 1 presents five different types of lectins identified by these HMMs and present in more than one species, often within a larger taxonomic group of Rhodophytes, highlighting conservation. The complete results, including genome and protein accession numbers, can be found in Supplementary Tables.

Most HMMs did not yield significant matches with any of the 15 selected Rhodophytes, suggesting these are absent in the investigated red algae: PF14200 (RicinB_lectin_2), PF02140 (Gal_Lectin), PF00337 (Gal-bind_lectin), PF01453 (B_lectin), PF07472 (PA-IIL), PF09222 (Fim-adh_lectin), PF09264 (Sial-lect-inser), PF14517 (Tachylectin), PF11476 (TgMIC1), PF16458 (Beta-prism_lec), PF18560 (Lectin_like), PF17882 (SBD), PF13868 (TPH), PF21595 (CCL2-like), PF21708 (Glyco_hydro_59_C), PF00354 (Pentraxin), PF00187 (Chitin_bind_1), and PF14299 (PP2 or phloem lectins). Nevertheless, various lectin HMMs did yield unexpected results. For example, the number of unique lectins uncovered seems to be correlated with the algae Family, with the Bangiaceae and Gracilariaceae Families giving many more lectin hits. Also, several lectin types were only observed within certain Families (Table 1). Finally, the OAAH-family lectin sugar-binding domain (SBD), detectable by the HMM with Pfam number PF17882, was found in *Kappaphycus*, *Eucheuma*, and *Solieria* (belonging to the order Gigartinales) for which no high-quality and annotated genomes are currently available.

Multiple CTLs are present in *Neopyropia yezoensis* and *Porphyra umbilicalis* of the Bangiaceae family, as well as one to three CTLs in *Gracilaria domingensis* and *Gracilariopsis chorda* of the Gracilariaceae family of Rhodophytes. CD-search furthermore revealed that in Rhodophyte CTLs, the protein domain architecture includes a von Willebrand factor (vWFA) domain and an HK sensor domain adjacent to the CLECT domain. Some C-type lectins in the nematode *Caenorhabditis elegans* also feature a vWFA domain, and the genes coding for these proteins are up-regulated in response to pathogens [63]. However, the combination of vWFA, HK sensor, and CLECT domain seems to be limited to Rhodophyta (Porphyra), Viridiplantae (Chloropicon, Ostreobium, Bryopsis), and Cryptophyceae (Rhodomonas), as indicated by CDART, the Conserved Domain Architecture Retrieval Tool [64]. In general, CTLs, FTLs, GTLs, X-type lectins, and pentraxins have been uncovered more abundantly in marine animals than other lectin classes, possibly explaining a larger number of CTLs discovered in Rhodophytes [33]. The presence of these lectins may be related to the interaction of seaweeds with diverse epiphytic bacteria and other organisms in the sea environment [65], as well as their role in sensing damaged tissues resulting from herbivory and other injuries caused by environmental factors [28,66].

LTLs are a diverse class of lectins and are widely used in biomedical and analytical applications due to their unique tertiary structures, which distinguish them from other lectins. At least 11 of the 15 Rhodophyte genomes contain one or two LTLs (Pfam: PF00139 and PF03388), which are similar either to plant concanavalin A-like lectins (Pfam: PF00139) or to human ERGIC-53 and VIP36 (Pfam: PF03388). ERGIC-53 and its related proteins, ERGL and VIP36, share an LTL folding motif and are type I membrane proteins involved in vesicular protein transport within the secretory system. No specific GTLs or pentraxins were uncovered in this genomic analysis.

Multiple JTLs were found in *Gracilaria domingensis* and *Gracilariopsis chorda*, as opposed to a complete absence of these lectins in other Rhodophyte Families. Some of these were annotated as nattering-like proteins and, indeed, show a domain architecture of a jacalin-like domain with an aerolysin domain, which is typically found in one of the natteries [67]. The jacalin-related β-prism I domain is widely distributed across all living organisms. It can exist as an individual lectin, such as for jacalin or griffithsin, or as a domain associated with other protein components in complex, multidomain proteins from bacteria, fungi, and plants. The mannose-specific lectins from red algae are phylogenetically more closely related to jacalin-related lectins from higher plants compared to other jacalin-related lectins of fungal or bacterial origin [68]. Malectins were abundantly found in six of the annotated Rhodophyta genomes, including all three species of the Florideophyceae class and some, but not all, species of the Bangiophyceae class. Notably, in the Bangiaceae family members *Neopyropia yezoensis* and *Porphyra umbilicalis*, there are over 20 malectin domain-containing carbohydrate-binding proteins. While some of these proteins contain only a malectin domain, most also include a so-called YliI domain, named after the *Escherichia coli* yliI gene, which encodes a glucose/arabinose dehydrogenase, and an Immunoglobulin-like fold, Plexins, Transcription factors (IPT) domain. IPT domains are also known as Transcription factor ImmunoGlobin (TIG) domains. This specific domain architecture appears to be unique to Rhodophyta and some bacteria based on CDART. The malectin domain, while structurally conserved and prevalent across prokaryotes, plants, and animals, exhibits distinct domain organizations and functions within each group [25]. In animals, malectin is a membrane-anchored carbohydrate recognition domain (CRD) located in the endoplasmic reticulum (ER), where it binds N-linked glycans during glycoprotein biosynthesis. They are up-regulated under ER stress and interact with ribophorin I, contributing to endoplasmic reticulum quality control (ERQC) and the unfolded protein response (UPR) [54]. In plants, malectins perform similar functions [69], and their CRD is found on the cell surface and is linked to a cytoplasmic kinase domain. In bacteria, malectin domains are associated with glycohydrolase domains. Our genome analysis indicates that these proteins are also present in seaweeds, and their abundance probably reflects that as for animals and land plants, they play diverse biological roles in seaweed development and are key players in the responses of seaweeds to environmental stresses, including fluctuations in temperature, salinity, irradiance, and the physical impacts of wave action.

The RTL superfamily is categorized as a single domain (Pfam: PF14200) or a tandem-repeat domain (Pfam: PF00652) [57]. R-type lectins are present in fungi, protists, invertebrates, and vertebrates. Almost all of them have the same Q-x-W sequence and bind to Gal, GalNAc, and Man. A ricin B-type lectin is present in species of the Cyanidiaceae family (*Cyanidiococcus yangmingshanensis*, *Cyanidioschyzon merolae*, *Cyanidium caldarium*). Although it is currently annotated as a hypothetical protein in these species, it combines a PGAP4-like domain with a ricin-like domain. The PGAP4-like domain shows similarity to animal post-GPI attachment to protein factor 4 (PGAP4), which has been shown to be a Golgi-resident GPI-GalNAc transferase. Another similar ricin B type lectin, annotated as N-acetylgalactosaminyltransferase, was found in *Porphyridium purpureum* of the Porphyridiaceae family and in the Gracilariaceae family of the class Florideophyceae (*Chondrus crispus*, *Gracilaria domingensis*, *Gracilariopsis chorda*). N-acetylgalactosaminyltransferases have chimeric-form RTL structures consisting of an N-terminal glycosyltransferase family A (GT-A) catalytic subdomain and a C-terminal RTL domain. The batch CD-search indeed confirmed that in these four species, the proteins combined a N-acetylgalactosaminyltransferase (GalNAc-T) domain followed by a ricin B lectin domain (PF00652). These enzymes are widely distributed across vertebrates, invertebrates, and protists. However, they have not yet been identified in bacteria, plants, or fungi [57].

Remarkably, an HTL detected by the Hidden Markov Model with Pfam number PF09458, listed in Table S2, was only found in *Gracilaria domingensis* and not in any other Rhodophyte genomes. BLASTp analysis of this H-type lectin (GenBank: KAI0563490) revealed homologs only in the Dikarya subkingdom of fungi and in certain bacteria, with the closest match being a metagenome-assembled genome (MAG) of *Ktedonobacteraceae* bacterium (GenBank: HZU01106).

## 4. Applications of Rhodophyta Lectins in Biotechnology and Medicine

Lectins from Rhodophyta exhibit a remarkable diversity in carbohydrate-binding specificities and structures, contributing as valuable molecules in various biological and pharmaceutical contexts. From the 1960s to 1990s, researchers extensively studied extracts from Rhodophyta isolating glycoproteins that were often referred to as agglutinins or hemagglutinins due to their capacity to bind selectively to carbohydrate moieties on cell surfaces, including erythrocytes [21,70,71,72]. Over time, researchers recognized that these carbohydrate-binding glycoproteins shared functional and structural characteristics of lectins, catalyzing a shift in terminology and advancing the understanding of their roles as specialized carbohydrate-binding proteins [73,74].

The first report of hemagglutination activity in seaweed was documented by Boyd et al. [21]. In this study, among 24 aqueous extracts tested, six brown algae and one cyanobacterium demonstrated the ability to agglutinate erythrocytes from blood groups A and O. Additionally, one green alga and two brown algae agglutinated erythrocytes from all blood groups, while one red alga specifically agglutinated erythrocytes from group A. Nevertheless, the first red seaweed lectin to be purified and partially characterized was that of *Ptilota plumosa*, which was only performed in 1977 [75].

**Figure 2:**
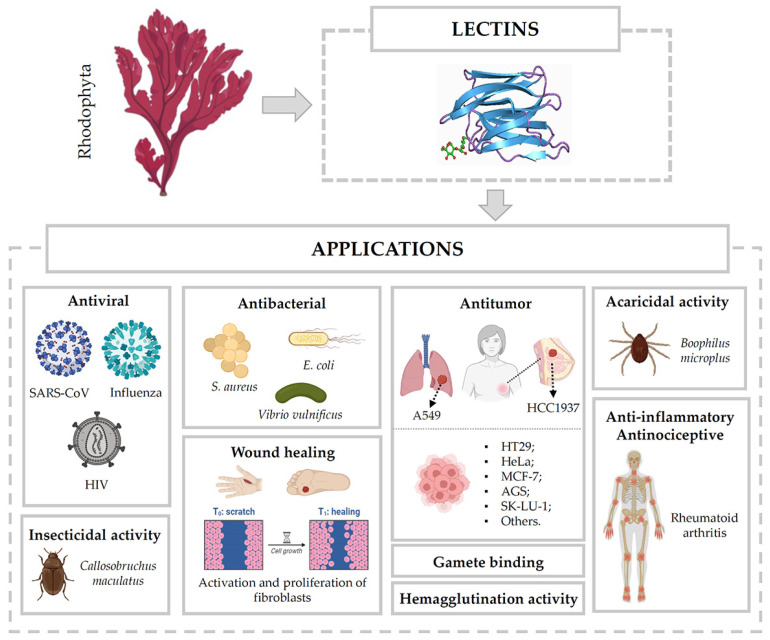
Applications of Rhodophyta lectins. This figure illustrates the versatility of lectins derived from Rhodophyta as bioactive molecules with significant potential in biomedical, pharmaceutical, agricultural, and biotechnological applications.

As research advanced, it became evident that Rhodophyta lectins possessed far more potential than their hemagglutination properties alone. These lectins, typically low-molecular-weight and stabilized by multiple disulfide bonds, are minimally immunogenic and structurally robust within host systems. They exhibit high specificity for complex glycoconjugates, particularly those found on cell surfaces, allowing for targeted interactions that can be leveraged in numerous biological applications. Due to these unique properties, red algal lectins have emerged as promising agents in antiviral (against SARS-CoV, influenza virus, and HIV), antibacterial, anti-inflammatory, and antitumor therapies, as well as in wound healing and tissue regeneration, among other therapeutic domains (see Figure 2 and Table 2).

Depending on their structural scaffold, mannose-specific seaweed lectins are categorized into five distinct, structurally related lectin families [81]: the Griffithsin lectin family (β-prism I scaffold), the *Oscillatoria agardhii* agglutinin homolog (OAAH) lectin family (β-barrel scaffold), the legume lectin-like lectin family (β-sandwich scaffold), the *Galanthus nivalis* agglutinin (GNA)-like lectin family (β-prism II scaffold), and the MFP2-like lectin family (MFP2-like scaffold). Griffithsin (GRFT) from the red alga *Griffithsia* sp. exhibits significant antiviral properties, positioning them as promising candidates for therapeutic development [52,101,102]. GRFT is a carbohydrate-binding lectin with a relative molecular mass of 13 kDa that targets high-mannose glycans on viral envelope glycoproteins, effectively inhibiting the binding and entry of viruses such as HIV and SARS-CoV into host cells. This antiviral mechanism is based on GRFT’s ability to interact with viral glycoproteins like gp120 and gp41 in HIV, blocking the initial stages of viral infection at extremely low effective concentrations (EC50 ranging from 0.043 to 0.63 nM). Additionally, other mannose- and high-mannose-binding lectins have shown promising antiviral activities. KAA-2 from *Kappaphycus alvarezii* demonstrates broad-spectrum anti-influenza activity, effectively inhibiting infection by a range of influenza strains, including H1N1, with EC50 values in the low nanomolar range [95]. KAA-2’s oligosaccharide-binding profile aligns closely with that of ESA-2 from *Eucheuma serra* (another red alga) and OAA from *Oscillatoria agardhii* (cyanobacterium), both of which strongly inhibit HIV infection by directly binding to the HIV envelope glycoprotein gp120 [103].

Another notable lectin is GCL from *Grateloupia chiangii*, a ~25 kDa protein with potent antiviral effects, particularly against herpes simplex viruses (HSV-1 and HSV-2), where it demonstrated an EC50 range of 1 to 20 nM, though it was less effective against influenza virus (PR8, HK, and Lee) [80]. These findings collectively underscore the diverse antiviral mechanisms of red algal lectins, positioning them as versatile agents in antiviral therapeutics against pathogens that rely on glycosylated envelope proteins for cell entry.

Rhodophyta lectins also exhibit significant anti-inflammatory potential through various immune-modulating and cytokine-influencing mechanisms. *Pterocladiella capillacea* lectin (PcL) has demonstrated potent anti-inflammatory and analgesic effects in animal models, notably by reducing neutrophil migration and carrageenan-induced paw edema [99]. Its peripheral anti-nociceptive activity, effective in reducing pain in formalin-induced models, appears to operate through inhibition of inflammatory mediators rather than central nervous system pathways, suggesting a peripheral mechanism of pain relief. *Caulerpa cupressoides* lectin (CcL) has shown similar anti-inflammatory and pain-reducing effects, further highlighting Rhodophyta lectins’ peripheral efficacy in inflammation-related pain relief [97]. These lectins also modulate cytokine responses, shifting immune reactions toward Th2 dominance [94]. In BALB/c mice, lectins from *Solieria filiformis* (SfL) and *Pterocladiella capillacea* (PcL) increase levels of IL-10, a key anti-inflammatory cytokine, while promoting nitric oxide (NO) production, which together contribute to their immunomodulatory and anti-inflammatory properties. Moreover, the lectin from *Amansia multifida* Lamouroux (AmL) suppresses proinflammatory cytokines, including TNF-α and IL-1β, and reduces oxidative stress markers like glutathione (GSH) in inflammatory models, effectively lowering leukocyte infiltration and edema [77]. These effects offer significant protection against inflammation-induced oxidative damage. SfL has hemagglutinating activity against bacterial strains of *Escherichia coli* and *Staphylococcus aureus*, as well as inhibiting the formation of biofilms by *E. coli*. These results suggest that the lectin may interact with components of the extracellular polymeric matrix. In the context of healing, SfL also stands out for its anti-inflammatory effects, promoting a reduction in the inflammatory response and stimulating the activation and proliferation of fibroblasts, indicating its therapeutic potential in tissue repair processes [76]. With a molecular weight of roughly 9.19 kDa and 90 amino acid residues, the lectin *Hypnea cervicornis* agglutinin (HCA) from *Hypnea cervicornis* J. Agardh was evaluated for its anti-inflammatory activity. Research suggests that HCA could be a therapeutic option for treating human rheumatoid arthritis. This is because HCA has shown anti-inflammatory and anti-nociceptive effects in rat models of zymosan-induced arthritis when given as a treatment after the condition’s onset. The anti-nociceptive action of HCA is attributed to a mechanism that includes the stimulation of the cyclic GMP signaling pathway, inhibition of leukocyte inflow, and gene expression of pro-inflammatory factors like TNF-α and iNOS [79].

The anticancer applications of Rhodophyta-derived lectins stem from their specificity in binding high-mannose-type N-glycans, which are frequently overexpressed on the surface of cancer cells. This carbohydrate specificity allows these lectins to interact selectively with tumor cells, leading to the induction of apoptosis and inhibition of cell proliferation. Noteworthy examples of these red algal lectins include *Eucheuma serra* agglutinin (ESA), *Solieria filiformis* lectins (Sfl), *Kappaphycus alvarezii,* and *Kappaphycus striatus* lectins, which demonstrate structural and functional characteristics with distinct anticancer capabilities [8,78,104,105]. ESA has demonstrated notable apoptotic activity in colon cancer cells both in vitro and in vivo [104,105]. Preclinical studies in mouse models have shown that ESA administration effectively reduces tumor size while exhibiting minimal cytotoxicity to non-cancerous cells. ESA induces apoptosis through caspase-3 activation and phosphatidylserine translocation to the outer cell membrane, marking the activation of intrinsic apoptotic pathways. These molecular markers underscore ESA’s promise as a targeted agent in cancer therapy by specifically triggering apoptosis in malignant cells. Lectins from *Solieria filiformis* and *Kappaphycus alvarezii* show similar apoptotic mechanisms. The Sfl lectins from *S. filiformis* have been reported to activate caspase pathways, resulting in apoptotic cell death in breast cancer cells [86]. These lectins possess multiple tandem repeats of carbohydrate-binding domains, which contribute to their high affinity and selectivity for specific high-mannose glycans. Additionally, the newly identified lectin Siye from *Kappaphycus alvarezii* has shown potent antitumor activity, binding selectively to cancer-associated glycans without eliciting agglutination in erythrocytes, a property that favors its systemic administration with minimal adverse immune responses [8]. Siye has been shown to initiate both early and late apoptotic stages in cancer cell lines, including human lung carcinoma cells (A549) and human breast adenocarcinoma cells (HCC1937). Sequence analysis has revealed a high degree of homology between Siye and previously identified lectins KAA-1 and KAA-2, underscoring the potential evolutionary advantage of these lectins in binding high-mannose structures. Furthermore, recent studies on *Kappaphycus striatus* have identified another lectin with anticancer properties that share significant sequence homology with other high-mannose binding lectins, including ESA-2, KAA-1, KAA-2, and Sfl-1 and Sfl-2 [78]. This structural similarity suggests that *Kappaphycus striatus* lectin (KSL) may possess similar binding characteristics, enhancing its affinity for high-mannose-type glycans on tumor cells and facilitating targeted apoptotic effects.

Marine red algae, particularly through their extracts and lectins, show strong antibacterial effects against specific pathogenic *Vibrio* species [91,106]. Saline and aqueous ethanol extracts from species like *Eucheuma serra* and *Pterocladia capillacea* demonstrate significant antibacterial activity against *Vibrio vulnificus*, a fish pathogen, though they do not affect *Vibrio neresis* [106]. Lectins from *E. serra* (ESA) and *Galaxaura marginata* (GMA) also strongly inhibit *V. vulnificus*, highlighting the selectivity of these red algal compounds. The antibacterial action of these extracts appears to rely on carbohydrate interactions, as they can be reduced by pre-treatment with specific sugars and glycoproteins. Moreover, environmental conditions, such as light intensity, nutrient levels, and moderate temperatures, enhance the production of antibacterial metabolites in *P. capillacea*. Additionally, lectins from *Eucheuma denticulatum*, known as EDAs, exhibit targeted antibacterial effects against the shrimp pathogen *Vibrio alginolyticus*, showing a specific binding affinity for branched oligomannosides on glycoproteins [91]. This binding specificity underpins their selective antibacterial function, which is disrupted in the presence of yeast mannan—a glycoprotein with high-mannose N-glycans—indicating that EDAs act by attaching to specific receptors on bacterial surfaces. Importantly, the lectin from *Solieria filiformis* extends these antibacterial capabilities to human pathogens, selectively inhibiting certain Gram-negative bacteria, including *Serratia marcescens*, *Salmonella typhi*, *Klebsiella pneumoniae*, *Enterobacter aerogenes*, *Proteus spp*., and *Pseudomonas aeruginosa* [107]. Significant reductions were observed in *E. aerogenes* and *P. aeruginosa* cell counts, which dropped to 0.3 × 10^9^ and 0.5 × 10^9^ CFU/mL, respectively, compared to control levels of 3.2 × 10^9^ and 4.9 × 10^9^ CFU/mL. The antibacterial effects of *S. filiformis* lectin are likely mediated through interactions with surface mannan on these Gram-negative bacteria, underscoring the lectin’s potential as a targeted antimicrobial agent. Notably, the lectin did not affect other bacteria, such as *Escherichia coli*, *Salmonella typhimurium*, *Bacillus subtilis*, and *Staphylococcus aureus,* suggesting its selective action may depend on the presence of specific surface carbohydrates, thereby offering a promising avenue for antibacterial applications against human pathogens while minimizing off-target effects. This selective mechanism enhances the biomedical value of red algal lectins, particularly in the development of specialized antibiotics targeting harmful human pathogens.

Lectins are glycoproteins that selectively bind to carbohydrates on the cell surface, a process called agglutination causes the cells to cluster together. This phenomenon is particularly evident in erythrocytes (red blood cells), where lectin–carbohydrate interactions on the cell membrane promote the formation of visible cell aggregates. The specificity of agglutination depends on the molecular structure of the carbohydrates and the lectins involved, making it a valuable tool in blood typing and cellular identification research. Different Rhodophyta strains have been reported in the literature as producers of lectins with hemagglutinating activity, including species such as *Acanthophora spicifera* [108], *Asparagopsis taxiformis* [108], *Boodlea coacta* [109], *Carpopeltis flabellate* [109], *Enantiocladia duperreyi* [70], *Galaxaura filamentosa* [108], *Gracilaria bailiniae* [108], *G. bangmeiana* [108], *G. changii* [108], *G. eucheumatoides* [108], *G. fisheri* [53], *G. salicornia* [108], *G. verrucosa* [72], *Grateloupia filicina* [108], *Halymenia maculate* [108], *Hypnea boergesenii* [108], *H. cervicornis* [110], *H. japonica* [109], *H. nidulans* [108], *H. valentiae* [108], *Laurencia obtuse* [108], *Meristiella echinocarpa* [86]*, Palmaria palmata* [71], *Solieria robusta* [109], *Tichocarpus crinitus* [100,111], and *Vidalia obtusiloba* C. Agardh [112]. This hemagglutination activity in Rhodophyceae suggests that their lectins exhibit a high affinity for specific sugars on erythrocyte surfaces, underscoring their potential for studies on cell interactions and possible biotechnological applications.

The lectin isolated from *Gracilaria cornea* has shown promise as a strategy for controlling cattle ticks (*Boophilus microplus*) due to its acaricidal effects. This lectin significantly reduced the weight of females after the oviposition period, the total mass of eggs produced, the hatching period of the eggs, and the mean survival time of the larvae. These results highlight the potential of lectins as natural and effective alternatives for managing livestock parasites, reducing reliance on synthetic acaricides [113]. Similarly, in the context of lectins with insecticidal properties, a lectin isolated from *Gracilaria ornata* demonstrated significant inhibitory effects on the development of *Callosobruchus maculatus* larvae in cowpea seeds, further underscoring its potential as a biotechnological tool for pest management [114].

The results highlighted the therapeutic potential and wide range of lectins identified in Rhodophyta. These lectins exhibit different biological activities, including antiviral, antibacterial, anti-inflammatory, anti-nociceptive, and antitumor properties, emphasizing their potential for new biomedical applications. The capacity of red algal lectins to specifically bind to glycan structures, such as high-mannose N-glycans, plays a key role in their mechanism of action across different disease contexts. This glycan-binding specificity facilitates therapeutic approaches for cancer and infectious diseases and broadens their potential application in inflammation management, for example. Nevertheless, further research is still essential to understand the mechanisms that drive these lectins’ bioactivity and advance their development into clinically applicable therapeutics.

## 5. Conclusions and Future Perspectives

This review of the literature and publicly available genomes reveals the presence of at least six distinct types of lectins across multiple Rhodophyte species. These lectins, which include ricin B or R-type lectins, jacalin-like lectins, C-type lectins, leg- or L-type lectins, malectins, and OAAH-family-type lectins, demonstrate significant conservation within specific taxonomic groups.

Rhodophyta lectins exhibit a remarkable diversity in carbohydrate-binding specificities and structures, making them valuable in various biological and pharmaceutical contexts. Historically, these lectins were extensively studied and recognized for their carbohydrate-binding properties, leading to a deeper understanding of their roles. Notably, red algal lectins such as Griffithsin (GRFT) and KAA-2 have shown significant antiviral properties against viruses like HIV, SARS-CoV, and influenza by targeting high-mannose glycans on viral envelope glycoproteins. The mannose-binding lectin GCL from *Grateloupia chiangii* has also demonstrated significant antiviral activity against HSV and influenza, reinforcing the biomedical value of red algal lectins in developing specialized antibiotics and antiviral therapies.

These lectins also demonstrate potent anti-inflammatory and analgesic effects, making them promising candidates for inflammation control and pain management. Lectins like AmL from *Amansia multifida* and HCA from *Hypnea cervicornis* have shown notable anti-inflammatory properties, further supporting their therapeutic potential. Their ability to modulate cytokine responses and promote anti-inflammatory cytokines further highlights their potential in immune modulation therapies.

Rhodophyta-derived lectins have shown specificity for high-mannose-type N-glycans, often overexpressed on cancer cells, allowing them to selectively induce apoptosis and inhibit cell proliferation, thus presenting significant potential in cancer therapy. Lectins from *Solieria filiformis* and *Kappaphycus alvarezii* exhibit apoptotic mechanisms, activating caspase pathways to induce cell death in cancer cells. The newly identified lectin Siye from *Kappaphycus alvarezii* shows potent antitumor activity, binding selectively to cancer-associated glycans without causing erythrocyte agglutination, favoring its systemic administration with minimal immune response.

Additionally, marine red algae extracts and lectins demonstrate strong antibacterial effects against specific pathogenic *Vibrio* species and human pathogens, highlighting their potential as targeted antimicrobial agents. The unique properties of these lectins, including their stability, low immunogenicity, and high specificity, position them as versatile agents in antiviral, antibacterial, anti-inflammatory, and antitumor therapies, as well as in wound healing and tissue regeneration.

To advance our understanding of Rhodophyte genomics and lectin diversity, future research should focus on improving genome assembly and annotation quality. This includes generating high-quality, contamination-free genomes and employing advanced bioinformatics tools for accurate gene prediction and functional annotation. Additionally, expanding the genomic resources for underrepresented Rhodophyte species will provide a more comprehensive view of their lectome and its evolutionary significance. Further studies should also explore the functional roles of identified lectins in Rhodophytes, particularly their potential applications in biotechnology and medicine. Understanding the mechanisms underlying lectin-mediated processes could lead to novel therapeutic strategies and biotechnological innovations.

## Figures and Tables

**Table 1:** Lectins uncovered from fifteen high-quality genomes of Rhodophyta species by various Hidden Markov Models (PF-numbers). Abbreviations: Calcium-binding-type lectins (CTLs), Jacalin-type lectins (JTLs), Legume-type lectins (LTLs), and Ricin-type lectins (RTLs).

				Hidden Markov Models				
Class	Family	Species	Unique Lectin Hits	CTLs (PF00059)	JTLs (PF01419)	LTLs (PF00139, PF03388)	Malectins (PF11721, PF12819)	RTLs (PF00652)
Bangiophyceae	Bangiaceae	*Neopyropia yezoensis*	31	5	-	2	24	-
		*Porphyra umbilicalis*	19	3	-	2	14	-
	Cyanidiaceae	*Cyanidiococcus yangmingshanensis*	1	-	-	-	-	1
		*Cyanidiococcus yangmingshanensis*	1	-	-	-	-	1
		*Cyanidioschyzon merolae*	1	-	-	-	-	1
		*Cyanidium caldarium*	1	-	-	-	-	1
	Galdieriaceae	*Galdieria Partita*	1	-	-	1	-	-
		*Galdieria sulphuraria*	1	-	-	1	-	-
		*Galdieria sulphuraria*	1	-	-	1	-	-
		*Galdieria Yellowstonensis*	1	-	-	1	-	-
	Porphyridiaceae	*Porphyridium purpureum*	5	-	-	2	2	1
Florideophyceae	Gigartinaceae	*Chondrus crispus*	9	-	-	2	6	1
	Gracilariaceae	*Gracilaria domingensis*	19	1	5	1	10	1
		*Gracilariopsis chorda*	21	3	2	2	13	1
Stylonematophyceae	Stylonemataceae	*Rhodosorus marinus*	1	-	-	1	-	-

**Table 2:** Rhodophyte lectins and their bioactivities, described in the literature between 2010 and 2024.

Species	Lectin Name	Biological Activities	Ref.
*Kappaphycus alvarezii* *	Siye	Antitumor	[8]
*Solieria filiformis*	Sfl	Wound healing	[76]
*Amansia multifisa*	AmL	Anti-inflammatory	[77]
*Kappaphycus striatus* *	KSL	Anticancer	[78]
*Hypnea cervicornis*	HCA	Anti-inflammatory and anti-nociceptive	[79]
*Grateloupia chiangii*	GCL	Antiviral	[80]
*Agardhiella tenera*	ATA	Anticancer	[81]
*Bryoyhamnion seaforthii*	BSL	Anticancer and pro-healing	[81]
*Bryothamnion triquetrum*	BTL	Anticancer	[81]
*Eucheuma serra*	ESA	Anticancer and antibacterial	[68]
*Grithsia* sp.	GRFT	Antiviral	[82]
*Pyropia yezoensis*	C-type lectin; L-type lectin	Pattern Recognition Receptors	[83]
*Solieria filiformis*	SfL-1; SfL-2	Anticancer, antibacterial, anti-nociceptive and anti-depressant	[81]
*Bryothamnion triquetrum*	BtL	Anti-inflammatory	[84]
*Solieria filiformis*	SfL	Anti-depressant	[85]
*Solieria filiformis*	SfL-1; SfL 2	Anticancer	[86]
*Halimeda renschii*	HRL40	Anti-influenza	[87]
*Kappaphycus alvarezii*	KAA-1; KAA-2	Anti-HIV	[88]
*Solieria filiformis*	SfL	Anti-nociceptive and anti-inflammatory	[89]
*Aglaothamnion callophyllidicola*	Rhodobindin Variations	Drug delivery	[90]
*Eucheuma denticulatum*	EDAs	Antimicrobial	[91]
*Aglaothamnion callophyllidicola*	Rhodobindin	Gamete binding	[92]
*Aglaothamnion oosumiense*	AOL1	Gamete binding	[93]
*Solieria filiformis*	SfL	Anti-inflammatory	[94]
*Kappaphycus alvarezii*	KAA-2	Anti-influenza	[95]
*Kappaphycus striatum*	KSA-1; KSA-2; KSA-3	Hemagluttination	[96]
*Caulerpa cupressoides*	CcL	Anti-nociceptive and anti-inflammatory	[97]
*Hypnea cervicornis*	HCA	Anti-nociceptive	[98]
*Pterocladiella capillacea*	PcL	Anti-nociceptive and anti-inflammatory	[99]
*Tichocarpus crinitus*	TCL	Hemagglutination and mitogenic activity	[100]

* Lectins of *K. alvarezii* and *K. striatus* belong to the so-called *Oscillatoria agardhii* agglutinin (OAAH) family of lectins.

## Data Availability

The data used for this review are publicly available at NCBI. Accession numbers are listed in Supplementary Table S1.

## Supplementary Materials

The following supporting information can be downloaded at: https://www.mdpi.com/article/10.3390/md23010008/s1, Table S1: Overview of lectin domains identified in Rhodophyta genomes, categorized based on their Hidden Markov Model (HMM) profiles. Table S2: curated list of lectin proteins identified within the phycolectomes of Rhodophytes. Table S3: Hidden Markov Models (HMMs) used for lectin identification. Table S4: details of all Rhodophyte (red algae) genomes examined in review.
